# Prioritisation of Candidate Genes Underpinning COVID-19 Host Genetic Traits Based on High-Resolution 3D Chromosomal Topology

**DOI:** 10.3389/fgene.2021.745672

**Published:** 2021-10-25

**Authors:** Michiel J. Thiecke, Emma J. Yang, Oliver S. Burren, Helen Ray-Jones, Mikhail Spivakov

**Affiliations:** ^1^ Enhanc3D Genomics Ltd, Cambridge, United Kingdom; ^2^ Functional Gene Control Group, MRC London Institute of Medical Sciences, London, United Kingdom; ^3^ Institute of Clinical Sciences, Faculty of Medicine, Imperial College, London, United Kingdom; ^4^ Cambridge Institute of Therapeutic Immunology and Infectious Disease, Department of Medicine, University of Cambridge, Cambridge, United Kingdom

**Keywords:** COVID-19, GWAS (genome-wide association studies), enhancers and promoters, regulatory genome, 3D chromosomal architecture

## Abstract

Genetic variants showing associations with specific biological traits and diseases detected by genome-wide association studies (GWAS) commonly map to non-coding DNA regulatory regions. Many of these regions are located considerable distances away from the genes they regulate and come into their proximity through 3D chromosomal interactions. We previously developed COGS, a statistical pipeline for linking GWAS variants with their putative target genes based on 3D chromosomal interaction data arising from high-resolution assays such as Promoter Capture Hi-C (PCHi-C). Here, we applied COGS to COVID-19 Host Genetic Consortium (HGI) GWAS meta-analysis data on COVID-19 susceptibility and severity using our previously generated PCHi-C results in 17 human primary cell types and SARS-CoV-2-infected lung carcinoma cells. We prioritise 251 genes putatively associated with these traits, including 16 out of 47 genes highlighted by the GWAS meta-analysis authors. The prioritised genes are expressed in a broad array of tissues, including, but not limited to, blood and brain cells, and are enriched for genes involved in the inflammatory response to viral infection. Our prioritised genes and pathways, in conjunction with results from other prioritisation approaches and targeted validation experiments, will aid in the understanding of COVID-19 pathology, paving the way for novel treatments.

## Introduction

Patients with COVID-19 disease, caused by SARS-CoV-2 infection, show a broad range of symptoms and severity, from asymptomatic disease to fatal progressive respiratory failure (Hu et al., 2021). Several known epidemiological factors increase the risk of COVID-19 severity and mortality: old age, male gender and pre-existing medical conditions such as diabetes (Docherty et al., 2020; Huang et al., 2020). These factors, however, do not fully explain the variability and clinical outcome of COVID-19. Following the outbreak of the disease caused by a related virus, SARS, in 2002–2004, it was suggested that host genetic factors influence the clinical course and outcome of coronavirus infections (de Wilde et al., 2018). These findings have provided a motivation for a systematic identification of host genetic factors linked with COVID-19 susceptibility and severity using genome-wide association studies (GWAS). Most recently, the COVID-19 host genetic initiative (COVID-19 HGI) has joined up these efforts to produce GWAS meta-analyses in four case-control settings in ∼50 K patients and ∼2 M controls from 47 studies in total (at Release 5), thereby increasing the power and robustness of individual GWAS (COVID-19 Host Genetics Initiative, 2021).

Whilst GWAS have revealed the underpinning genetic components of many phenotypes (Buniello et al., 2019), translating the identified genotype-disease associations into actionable therapeutic targets has presented a major challenge. To a large extent, this is due to the fact that the absolute majority of GWAS variants map outside of the protein-coding and promoter regions of the genome and are instead enriched at distal DNA regulatory elements such as gene enhancers (Cano-Gamez and Trynka, 2020). Enhancers may localise long distances (hundreds of kilobasepairs) away from their target gene promoters and come into their physical proximity via 3D chromosomal contacts (Schoenfelder and Fraser, 2019; Ray-Jones and Spivakov, 2021).

Currently, 3D chromosomal contacts are typically measured by Hi-C, a chromatin proximity ligation technique using next-generation sequencing of the ligation junctions for detection (van Berkum et al., 2010). Theoretically, Hi-C makes it possible to map all pairwise genomic contacts in the genome at a restriction-fragment resolution. However, the high complexity of Hi-C sequencing libraries limits the practically achievable genomic coverage, leading to a reduced sensitivity and resolution of this method. This limitation can be effectively mitigated using Capture Hi-C (PCHi-C), which enriches Hi-C libraries prior to sequencing for fragment pairs that include, at least on one end, regions of interest, such as annotated gene promoters (Schoenfelder et al., 2018).

We previously developed COGS (Capture Hi-C Omnibus Gene Score), a formal statistical framework to capitalise on high-resolution chromosome conformation data such as PCHi-C to link GWAS variants with their putative target genes (Javierre et al., 2016; Burren et al., 2017). The COGS pipeline generates a Bayesian prioritisation score for each gene being causal for a given GWAS trait, with causal genes defined as those containing at least one causal variant in the coding region, promoter and/or promoter-interacting regions detected by PCHi-C.

Here we used COGS with our previously generated PCHi-C data in 17 primary blood cell types (Javierre et al., 2016) and in a SARS-CoV-2-infected lung carcinoma cell line (Ho et al., 2021) to prioritise candidate genes underpinning COVID-19 host genetic associations from COVID-19 HGI Host GWAS meta-analysis (COVID-19 Host Genetics Initiative, 2021). We prioritise 251 putative genes associated with SARS-CoV-2 infection and COVID-19 susceptibility and severity, the majority of which were not previously implicated in these traits, and characterise their expression patterns and functional annotations.

## Methods

### The COGS Prioritisation Pipeline

The COGS pipeline ​​(Javierre et al., 2016; Burren et al., 2017) takes GWAS summary data as input, fine-maps it using Wakefield synthesis ​(Wakefield, 2009) and aggregates the resulting posterior probabilities of a variant being casual across all promoter-interacting regions detected using PCHi-C data. It then uses LD block data to compute the probability that there is at least one causal variant in at least one gene-associated region, including promoter-connected fragments, promoter-proximal regions (the baited restriction fragment and its immediate flanking fragments) and/or the gene’s coding regions, under the assumption that there is at most one causal variant per LD block. COGS scores correspond to the estimated Bayesian probabilities of having at least one causal GWAS variant associated with a gene. Since COGS is primarily a ranking algorithm, the choice of the score threshold for gene prioritisation remains subjective in the absence of a gold standard. We used a COGS score threshold of 0.3 in reporting the numbers of prioritised genes and, where required, for downstream analyses, with data presented in the last section of Results confirming that our choice of threshold was appropriate for these purposes.

We ran the COGS pipeline using each of the four COVID-19 HGI GWAS datasets (release 5 excluding 23andMe data) using *HindIII-*based PCHi-C data in 17 human primary blood cell types ​(Javierre et al., 2016; Burren et al., 2017) and *DpnII-*based PCHi-C data (in 5 kb bins, with the baited fragments left unbinned) in A549-ACE2 cells​ at 0, 8 and 24 h after SARS-CoV-2 infection (Ho et al., 2021). The cell-type specificity of COGS scores may not be consistent with the expression patterns of the prioritised genes, while using COGS in a pooled setting across multiple samples increases the sensitivity of the analysis (Javierre et al., 2016). At the same time, the coverage and design of different PCHi-C datasets may have systematic effects on detected interaction signals (Freire-Pritchett et al., 2021). Therefore, COGS was run separately for data from each GWAS meta-analysis using a pool of promoter interactions with CHiCAGO ​​scores (Cairns et al., 2016) above 5 in at least one cell type in either dataset (Javierre: 707,583 interactions involving 21,102 baited promoter fragments; Ho: 43,265 interactions involving 9,955 baited promoter fragments). A minority of gene promoters were not baited in either PCHi-C capture system due to challenges in probe design and therefore not assayed in the respective systems. Therefore, their promoter-interacting regions could not be included in the analysis. To facilitate the analysis of their promoter-proximal variants, we generated “virtual baited fragments” for all annotated gene promoters. In addition, we included the coding variants of all annotated genes.

### Data Sources

COVID-19 HGI GWAS meta-analysis release 5 data were downloaded from https://www.covid19hg.org/results/r5/. This release jointly analysed nearly 50,000 COVID-19 cases and over two million controls by combining data from 47 studies across 19 countries. Details for each study are provided on the HGI website and in the consortium paper (COVID-19 Host Genetics Initiative, 2021). The CHiCAGO-processed PCHi-C data from Javierre and Ho were downloaded from OSF (https://osf.io/u8tzp) and GEO (accession GSE164533), respectively. LD block data were generated with LD-detect (Berisa and Pickrell, 2016) and downloaded from the software author’s website (http://bitbucket.org/nygcresearch/ldetect-data). Note that the LD block dataset did not include sex chromosomes, which were therefore excluded from COGS analysis. However, no strong association signals were detected on sex chromosomes in COVID-19 HGI GWAS, and therefore this limitation is unlikely to have missed strongly implicated genes.

The Javierre PCHi-C data are on GRCh37 assembly, and we used the GRCh37 versions of the COVID-19 HGI GWAS datasets, the original LD block data from Berisa and Pickrell and gene models from Ensembl GRCh37 Release 103 (https://grch37.ensembl.org) in the analysis. The Ho PCHi-C data are on GRCh38 assembly, and we used the GRCh38 versions of the COVID-19 HGI GWAS datasets, the lifted-over (GRCh37-to-38) LD block data and gene models from Ensembl GRCh38 Release 103. The results for each gene were linked between these analyses using Ensembl gene IDs as primary identifiers.

TPM-level gene expression data from GTEx and FPKM-level gene expression data from BLUEPRINT consortia were downloaded from GTEx portal (accession: phs000424. v8. p2) and EBI Gene expression atlas (accession: E-MTAB-3827), respectively. Gene sets of COVID-19 differentially expressed genes in multiple human cell types and tissues (106 conditions) were obtained from The COVID-19 Drug and Gene Set Library (https://maayanlab.cloud/covid19/) (Kuleshov et al., 2020). Differentially expressed genes in COVID-19 were obtained from Supplementary Table S2 in (Daamen et al., 2021) and the union of genes reported for peripheral blood mononuclear cells (PBMCs), lung tissue and bronchoalveolar lavage was taken. Hallmark gene sets were obtained from the Molecular Signature Database (https://www.gsea-msigdb.org/gsea/msigdb/index.jsp) (Liberzon et al., 2015).

### Gene-Level Manhattan Plots

Gene-level Manhattan plots were generated separately for each COGS run on a given GWAS and PCHi-C dataset using the R package ggplot2 (Wickham, 2016). Genes with COGS scores >0.3 were labelled in each locus. Multiple genes were labelled when there were several top-scoring genes with a very similar score, or lower-scoring genes with compelling biological functions. For simplicity, we did not label non-coding genes unless there were no prioritised protein-coding genes in the same locus.

### Comparison of COGS With Other Gene-Prioritisation Approaches

**FIGURE 1:**
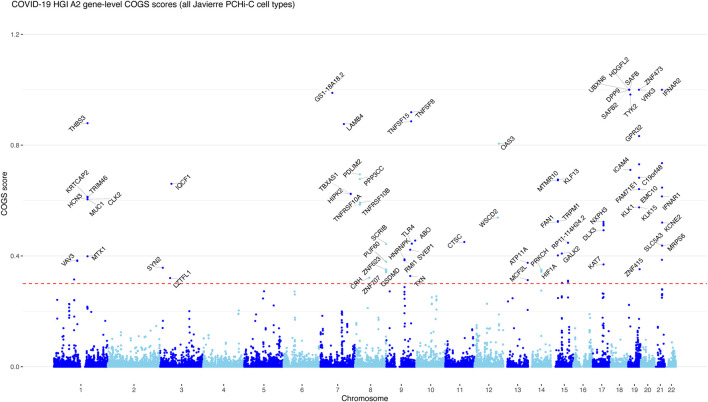
The COGS prioritisation scores of genes associated with A2 COVID-19 host GWAS trait. Gene-level Manhattan plot showing COGS scores generated based on A2 COVID-19 host GWAS data and PCHi-C data from COVID Javierrere et al. (2016). The top scoring genes (COGS scores >0.3) are labelled in each locus. Multiple genes are labelled when there are several top-scoring genes with a very similar score, or lower-scoring genes with compelling biological functions. For simplicity, non-coding genes are not labelled, unless there are no prioritised protein-coding genes in the same locus. See Supplementary Figures S1–S3 for the COGS gene-level Manhattan plots produced with the other three COVID-19 host GWAS traits based on PCHi-C data from Javierre et al. and Supplementary Figures S4–S7 for the prioritisation results based on PCHi-C data from Ho et al. (2021).

In the naive GWAS prioritisation approach, variants with nominal *p*-values below 10^−8^ were assigned to the nearest exon. The list of HGI-prioritised genes was taken from Figure 1 in the HGI consortium paper (COVID-19 Host Genetics Initiative, 2021). Genes outside of the regions highlighted in the Figure were defined as those whose TSSs mapped more than 1 Mb away from the lead variant. The list of TWAS- and SMR-prioritised genes was taken from Tables 2 and 3 in Baranova et al., 2021.

### Gene Expression Analysis

K-means clustering was performed on the scaled expression values of COGS-prioritised genes (score >0.3) in GTEx (TPM) and BLUEPRINT (RPKM) datasets using R package pheatmap with the number of clusters determined using the Silhouette and Elbow methods. GTEx analysis included 218 genes with detectable expression, and BLUEPRINT analysis focused on the 55 genes in the top 25% of expression in blood cells.

The GSEAPreranked analysis (Mootha et al., 2003; Subramanian et al., 2005) against COVID-19 differential expression signature gene sets used all genes returned by COGS, ranked by COGS score. Analysis was performed using the GSEA software (downloaded from www.gsea-msigdb.org) with default parameters and 1,000 permutations. Results were collated into a bubble plot using the R package ggplot2 (Wickham, 2016). Precision and recall analysis of COGS-prioritised genes versus COVID-19 differentially expressed genes (Daamen et al., 2021) was performed in R.

### Annotation of the Prioritised Genes

Kyoto Encyclopedia of Genes and Genomes (KEGG) pathway enrichment analysis (Kanehisa and Goto, 2000) used COGS-prioritised genes (score >0.3). The analysis was performed using the enrichKEGG function in the ClusterProfiler package (Yu et al., 2012) with an adjusted *p* value of 0.05. Significantly enriched pathways were visualised with KEGG mapper (Kanehisa and Sato, 2020). The GSEAPreranked analysis on Hallmark gene sets was run as for COVID-19-associated gene sets above.

## Results and Discussion

### Prioritisation of COVID-19 Host GWAS Genes Using PCHi-C Data

To prioritise candidate genes associated with COVID-19 susceptibility and severity, we integrated the worldwide meta-analysis data from the COVID-19 Host Genetics Initiative (COVID-19 HGI Release 5) (COVID-19 Host Genetics Initiative, 2021) with PCHi-C data using the COGS pipeline ​​(Javierre et al., 2016; Burren et al., 2017). COVID-19 HGI divided the patients into three categories: A- very severe cases characterised by respiratory failure, B- all hospitalised cases, and C - all cases that tested positive for SARS-CoV-2 infection, generating a GWAS meta-analysis for the following four traits: A2 (very severe cases vs population), B1 (hospitalised vs non-hospitalised Covid-19 patients), B2 (hospitalised patients vs population) and C2 (confirmed Covid-19 vs population) (COVID-19 Host Genetics Initiative, 2021).

We first used high-coverage PCHi-C data in 17 human primary blood cell types (Javierre et al., 2016), including endothelial progenitors, as the source of 3D chromosomal contacts for COGS. We prioritised 234 genes with COGS scores above 0.3 across the four GWAS, of which 37 had scores above 0.75. More than half of the prioritised genes (122/234) were detected from A2 GWAS, consistent with the number of significant variant-trait associations in this study. A total of 78 genes were uniquely prioritised from A2 and not the other three GWAS. Including B2 in the analysis contributed an additional 71 genes, followed by B1 and C2 (26 and 15 additional genes, respectively).

We expressed the prioritisation analysis results in the form of gene-level Manhattan plots (Figure 1 and Supplementary Figure S1–S3), which showed that clusters of adjacent genes were often prioritised jointly. In some cases, this was due to two promoters sharing the same PCHi-C baited fragment (e.g., *VRK3* and *ZNF473*). However, multiple genes may genuinely share GWAS variant-containing enhancers (Ray-Jones and Spivakov, 2021). Therefore, we have avoided further “fine-mapping” of COGS associations to the top-scoring gene in each peak.

We next used PCHi-C data from our recent analysis of a SARS-CoV-2 infected lung cell line (ACE2-expressing A549 cells) and uninfected controls (Ho et al., 2021). This experiment used a different PCHi-C design, based on *DpnII* and analysed in 5 kb bins (outside of the baited promoter regions that were left unbinned), as opposed to *HindIII* in the Javierre et al. blood cell analysis. This analysis returned 60 prioritised genes with COGS scores above 0.3, of which 13 had scores above 0.75. The gene-level Manhattan plots for this analysis are shown in Supplementary Figure S4–S7. The lower number of genes compared with the blood cell data is expected given the lower sequencing coverage and the smaller number of cell types profiled in this experiment. Over 70% (43/60) of the genes prioritised using this dataset (COGS score >0.3) also had scores above 0.3 in the blood cell-based analysis, indicating that the results of COGS prioritisation show a significant degree of consistency across different cell types and PCHi-C array designs.

Overall, 251 unique genes were prioritised based on four GWAS and two PCHi-C datasets at COGS score >0.3. The full results for all genes with their associated COGS scores are presented in Supplementary Table S1.

### Comparison of COGS With Other Gene-Prioritisation Approaches

#### Comparison With Nearest-Exon Variant-To-Gene Assignment

To compare the results of COGS prioritisation with a naive approach, we selected GWAS variants with nominal *p*-values below 10^−8^ (traditionally taken as a “genome-wide significance level” through the Bonferroni correction) and assigned them to the nearest exon. Across the four GWAS, this approach prioritised 45 genes, of which 11 were also prioritised by COGS at a score threshold of 0.3 (Supplementary Table S2). The genes prioritised by both approaches included 8/23 loci with coding variants (*ABO*, *DPP9*, *IFNAR2*, *KANSL1*, *LZTFL1*, *OAS1*, *OAS3*, *SLC6A20*), and 3/22 with non-coding variants mapped to their nearest exons (*AP000295.9, PDCL3P4, RP11-304F15.3*). Genes identified by the nearest-exon approach exhibited a wide range of COGS scores (Supplementary Figure S8A)*.* Unlike in the naive approach, COGS additionally incorporates data from promoter-interacting regions and has improved precision due to the use of statistical fine-mapping. Therefore, a limited overlap between these two approaches is expected.

#### Comparison With the COVID-19 HGI Gene Prioritisation Approach

The COVID-19 HGI consortium paper defined 13 genomic loci associated with infection or severe disease, and highlighted 47 putative gene targets across these loci. The genes highlighted in the consortium paper satisfied one or more of the following criteria: 1) being in close proximity to the lead variant, 2) overlapping disease-associated variants, 3) containing disease-associated coding variants (loss-of-function, missense), 4) being associated with an eQTL in LD with the lead variant, or 5) being prioritised by the OpenTargets V2G (Variant-to-Gene) algorithm (COVID-19 Host Genetics Initiative, 2021). HGI-prioritised genes showed a broad range of COGS scores (Supplementary Figure S8B), with 16 out of 47 HGI-prioritised genes showing scores above 0.3 (Supplementary Table S3A). For example, while HGI prioritised all three genes in the 2′-5′-Oligoadenylate synthetase (*OAS*) cluster, COGS prioritised *OAS3* (max COGS = 0.81) and *OAS1* (max COGS = 0.54), while *OAS2* had a subthreshold score (max COGS = 0.15).

At a COGS threshold of 0.3, a further 38/251 genes were prioritised within the 13 loci of genome-wide significance highlighted in the paper (Supplementary Table S3A). Notably, in the 21q22.11 locus we prioritised interferon A and B receptor subunit 1 (*IFNAR1*; max COGS = 0.91) in addition to the HGI-prioritised subunit 2 (*IFNAR2*; max COGS ∼1); the products of these two genes combine to form the type I interferon receptor (Piehler et al., 2012). In the 19q13.33 locus, the five HGI-prioritised genes had low COGS scores, whereas *BCAT2* (max COGS = 0.63) and *FTL* (max COGS = 0.41) were instead prioritised; of these, *FTL* (ferritin light chain) is reported to be anti-inflammatory (Zarjou et al., 2019). In the 19p13.3 locus, the Dipeptidyl peptidase 9 (*DPP9*) gene, which plays a key role in inflammasome regulation (Zhong et al., 2018), was confirmed with a COGS score of 1 (as well as two nearby non-coding genes: *DPP9-AS1* and *AC005783.1*). However, COGS also identified further seven distal gene targets including UBX domain protein 6 (*UBXN6*), which reportedly inhibits the degradation of COVID-19-implicated proteins IFNAR1 and TYK2 (Ketkar et al., 2021).

The remaining 197 out of 251 genes prioritised by COGS mapped outside of the 13 genome-wide significance loci (Supplementary Table S3B). These included such plausible candidates as LIF receptor (*LIFR*) and TNF receptor superfamily (*TNFSF*) members *10A/B, TNFSF8* and *15*, which have roles in cytokine signalling, as well as components of the PI3K/AKT signalling pathway (*LAMB4*, *THBS3*, *TLR4* and *YWHAE*), which was recently proposed as a therapeutic target in COVID-19 (Khezri, 2021).

#### Comparison With a Multiomics-Based Prioritisation

A recent study (Baranova et al., 2021) tested the colocalisation of COVID-19 HGI GWAS signals with expression and methylation quantitative trait loci using a combination of transcriptome-wide association study (TWAS) and Summary-based Mendelian randomisation (SMR). This approach prioritised 14 genes, five of which (*IFNAR2, MGC57346/LINC02210*, *OAS1, OAS3,* and *TYK2*) were also prioritised by COGS (score > 0.3). The remaining 9/14 genes had COGS scores ranging from zero to 0.249 (Supplementary Table S4).

Overall, while COGS analysis has confirmed the prioritisation of several genes found by the nearest-exon and alternative priorisation approaches, it also revealed large numbers of further candidates. The summary of all four prioritisation approaches is presented in Supplementary Figure S8C and in Supplementary Table S4.

### Expression Patterns of COGS-Prioritised Genes

#### Tissue-specificity of the Prioritised Genes

**FIGURE 2:**
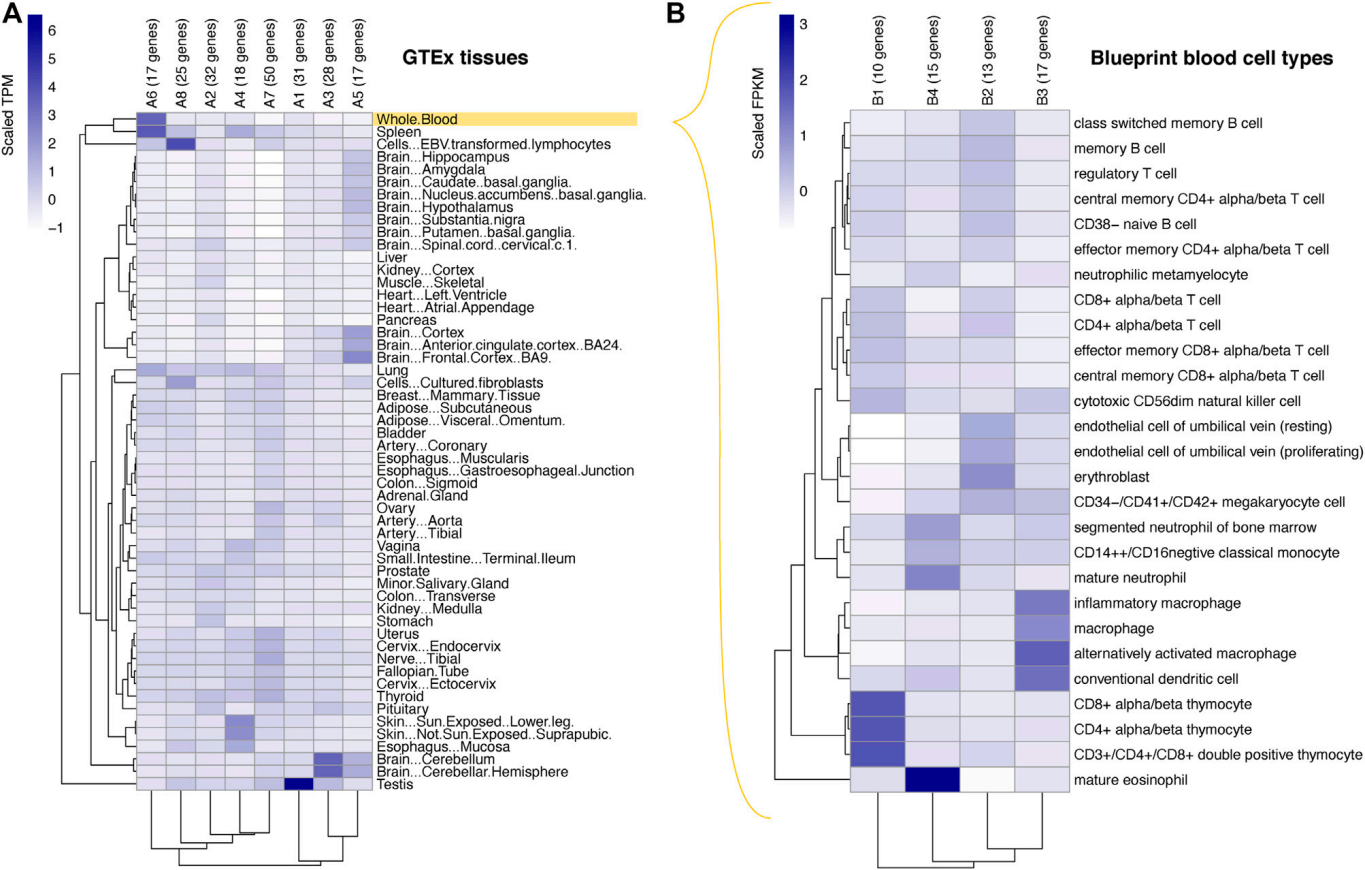
Expression patterns of the prioritised genes. Heatmaps showing the results of k-means clustering of COGS-prioritised genes (scores >0.3) based on their relative expression levels across the tissues profiled by the GTEx consortium **(A)** and across primary blood cell types profiled by the BLUEPRINT consortium **(B)**. Relative gene expression in **(A)** represents gene-level TPM values scaled across all GTEx genes, and in **(B)** gene-level RPKM values scaled across genes with the top 25% of expression in the BLUEPRINT dataset. Each cell in the heatmap represents a cluster, with the gene-to-cluster assignments listed in Supplementary Table S5A, B, respectively. Abbreviations: FPKM, fragments per kilobase of transcript per million mapped reads, TPM, transcripts per million.

To assess the gene expression patterns of the COGS-prioritised genes, we first took advantage of GTEx data across 54 non-diseased tissues (GTEx Consortium, 2020). In total, 218 genes were represented in this dataset. K-means clustering of scaled expression values segregated these genes into eight coherent clusters (Figure 2A; Supplementary Table S5A). Two large clusters (A6 and A8) containing 51 genes in total were characterised by their predominant expression in whole blood or EBV-transformed lymphocytes, respectively. This was expected from the involvement of well-characterised candidates such as *IFNAR1/2*, *OAS1/3* and *TYK2* in the immune function, as well as from the fact that the Javierre PCHi-C dataset was generated in blood cells. However, COGS also prioritised multiple genes active in other tissues, likely driven by promoter-proximal and coding variants**,** as well as promoter contacts shared across tissues. Genes in two other clusters (A3 and A5; 45 genes in total, including synapse-associated genes *SYN2*, *SYT3* and *SHANK1*) were predominantly expressed in different parts of the brain, consistent with the common neurological symptoms and evidence of brain damage following SARS-CoV-2 infection (Marshall, 2021). Somewhat surprisingly, cluster A1 (31 genes in total) contained genes showing high expression in testis, including sperm-associated calcium channel subunit *CATSPERG* and signal peptide peptidase *SPPL2C* active in spermatids. While SARS-CoV-2 is known to infect testis (Ma et al., 2021) and the male sex is a known risk factor for COVID-19 severity (Docherty et al., 2020; Huang et al., 2020), the exact role of these genes in COVID-19 pathology remains to be elucidated. The remaining three clusters (clusters A2, A4, A7; 100 genes in total) were characterised by broader expression patterns across multiple tissues, including the lung, gut, skin and vasculature.

To obtain a finer-grained view of the prioritised genes expression patterns in the blood, we studied their expression in 27 primary blood cell types using data from the BLUEPRINT consortium (Figure 2B; Supplementary Table S5B) (Chen et al., 2014). We restricted this analysis to 55 genes showing top 25% expression levels in the BLUEPRINT dataset. K-means clustering of their scaled expression values yielded four distinct clusters containing between 10 and 17 genes each, characterised by predominant expression in T lymphocytes (cluster B1), erythroblasts (B2), macrophages (B3) and mature eosinophils (B4), respectively. Examples of genes in these clusters include effectors of TNF (*TNFSF8,* B1; *TNFRSF10B*, B2), toll-like receptor (*TLR4,* B3) and interferon signalling (*IFNAR1* and *IFNAR2,* clusters B3 and B4, respectively).

Jointly, these results suggest the involvement of a broad range of blood cells and solid tissues in COVID-19 pathology.

#### Comparison With Reported COVID-19-Regulated Genes

**FIGURE 3:**
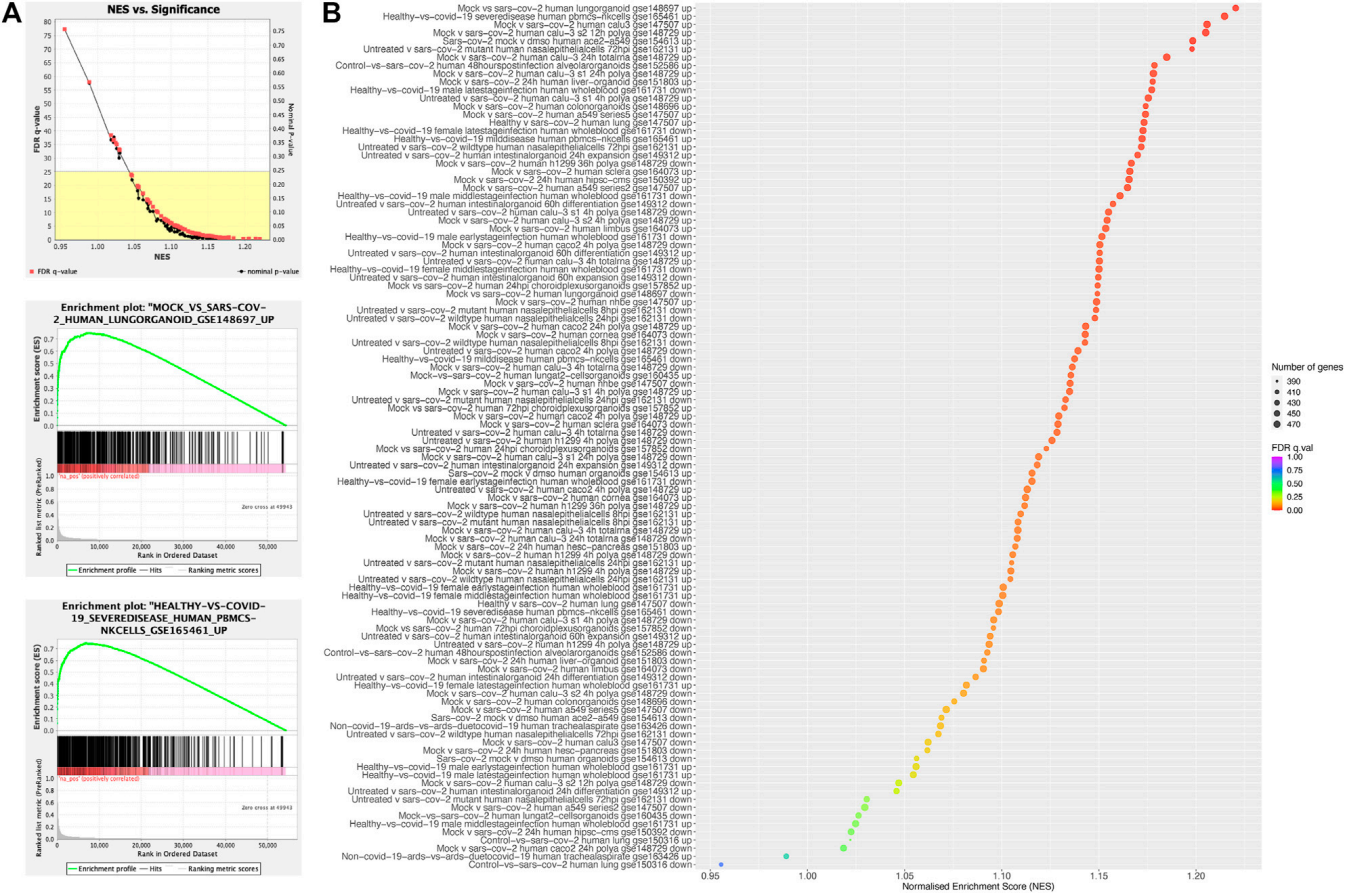
Enrichment of COGS-prioritised genes in COVID-19-response gene sets. Quantitative GSEA analysis using the COGS score for each gene against gene sets from the COVID-19 Drug and Gene Set Library. Diagnostic plots produced by the GSEA software demonstrate the relationship between the normalised enrichment score (NES) and measures of significance [**(A)**, top plot] and the enrichment across COGS scores for the gene sets with the top two NES scores (A, middle and bottom plots) **(B)** Bubble-plot showing results for all gene sets. The “up” and “down” suffixes indicated the direction of differential expression in COVID-19 for the gene set in question.

We asked if COGS preferentially prioritised genes that are known to change expression in response to COVID-19 infection. To address this question, we used the COGS scores of all annotated genes in a quantitative gene set enrichment analysis (GSEAPreranked) against 106 differential expression signature gene sets from The COVID-19 Drug and Gene Set Library (see Methods). All 106 COVID-19 gene sets had a positive Normalised Enrichment Score (NES), meaning that they were enriched at the top of the COGS-ranked gene list, with a mean FDR of 0.080 ± 0.125. This enrichment was significant at an FDR of 0.25 for 97 of the gene sets (Figure 3A; top plot and Supplementary Table S5C), indicating that the genes’ COGS scores positively associate with their differential expression in COVID-19. The top two sets, as ranked by the Normalised Enrichment Score (NES), were lung organoids infected with COVID-19 *in vitro* (top-ranking genes *IFNAR2, CHI3L2, OAS3*) and natural killer (NK) cells from individuals with severe disease versus healthy (top-ranking genes *DPP9, SAFB2, SAFB*) (Figure 3A middle and bottom plots, respectively). We noted that the sets achieving the highest NES tended to contain upregulated, rather than downregulated, genes (Figure 3B), suggesting a role of many underpinning variants in controlling gene induction in response to infection. Overall, these results provide additional validation that the COGS approach prioritises genes with relevance to COVID-19.

### The Biological Function of COGS-Prioritised Genes

**FIGURE 4:**
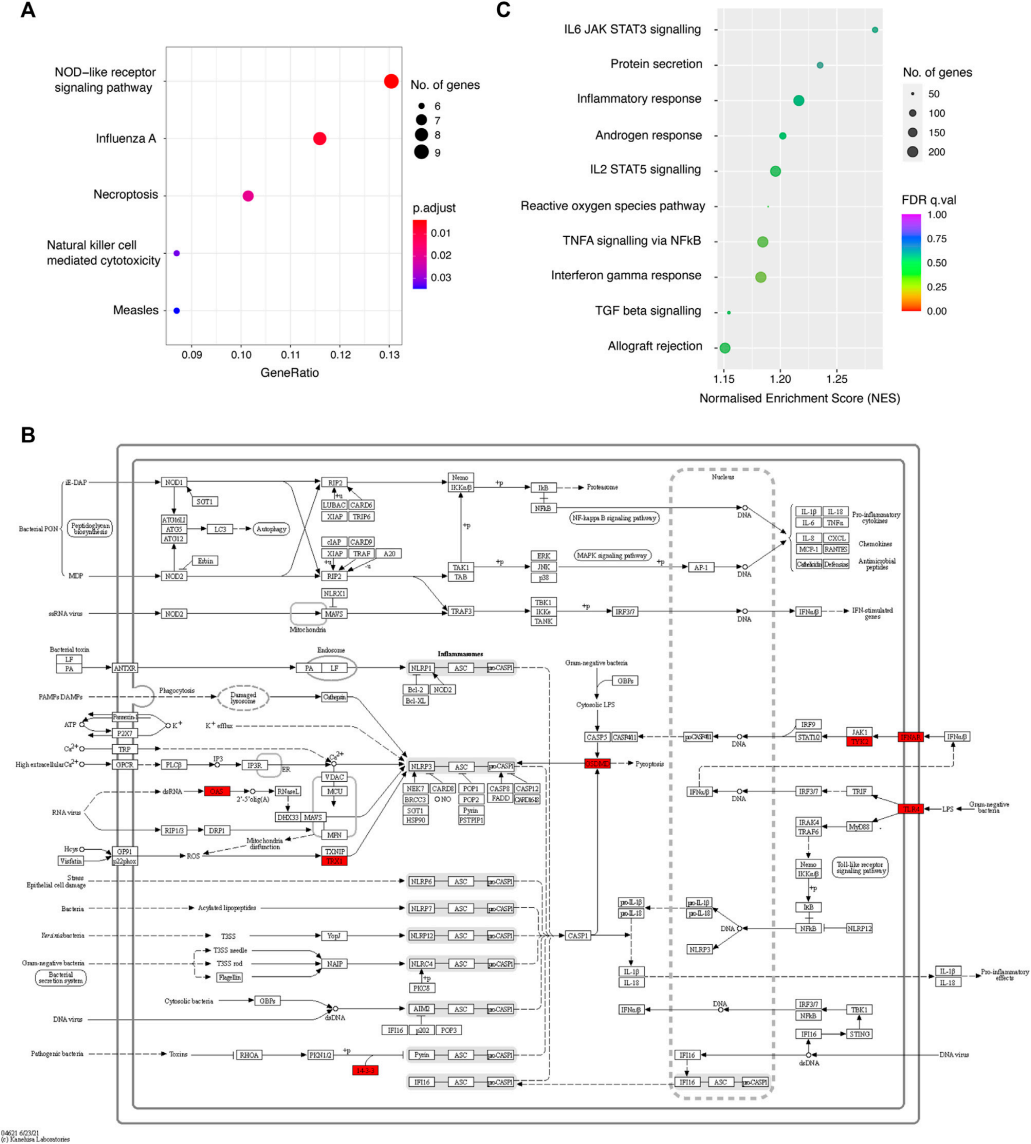
The biological functions of the prioritised genes. **(A)** Bubble plot showing the KEGG pathways enriched among COGS-prioritised genes (score >0.3). **(B)** Diagram of the NOD-like receptor signalling pathway, with the COGS-prioritised genes highlighted in red. **(C)** Bubble plot showing the results of a quantitative GSEA analysis using the COGS score for each gene against Hallmark gene sets.

To gain insight into the shared biological functions of the prioritised genes, we first performed KEGG pathway over-representation analysis (Figure 4A). We found that COGS-prioritised genes (max COGS score >0.3) were significantly enriched in pathways associated with response to influenza A and measles infection, as well as with inflammatory processes, including NOD-like receptor signaling (Figure 4B), necroptosis and natural killer cell-mediated cytotoxicity. These enriched annotations were driven by a total of 11 COGS-prioritised genes with a high overlap between individual pathways (*FTL*, *IFNAR1/2*, *OAS1*/*3*, *PPP3C*, *TLR4*, *TNFRSF10 A*/*B*, *TYK2*, and *VAV3*; Supplementary Table S6A). We note that all five enriched pathways are druggable according to the KEGG database (Kanehisa and Goto, 2000), creating potential opportunities for drug repurposing for COVID-19 treatment. For example, the NOD-like receptor signaling pathway alone is currently targeted by 14 drugs indicated for various inflammatory diseases, with one of these drugs, a selective IRAK4 inhibitor Zimlovisertib, undergoing a clinical trial for COVID-19-induced pneumonia (https://clinicaltrials.gov/ct2/show/NCT04575610).

To further increase the sensitivity of pathway enrichment analysis, we again performed quantitative GSEA based on the COGS scores, this time against 50 Hallmark gene sets from the Molecular Signatures Database. Although none of the Hallmark sets were significantly enriched at an FDR of 0.25 (Supplementary Table S6B), the top sets ranked by NES included relevant inflammatory processes such as IL-6 signalling by STAT3, IL-2 signalling by STAT5, TNF-α signaling via NFκB, IFN-γ response and TGF-β signalling (Figure 4C), highlighting the roles of individual COGS-prioritised genes in these processes.

Jointly, these results support the notion that genetically-determined variation in the inflammatory response to viral infection plays a key role in COVID-19 susceptibility and severity.

### Assessment of COGS Score Thresholds Based on Prioritisation of COVID-19-Differentially Expressed Genes

Since COGS-prioritised genes were enriched in gene sets associated with COVID-19 transcriptional response, we used this property to estimate the sensitivity and specificity of COGS analysis at a range of score thresholds. We focused on data from a recent COVID-19 host transcriptomics study reporting 11,170 differentially-expressed (DE) genes across PBMCs, lung and bronchoalveolar lavage samples (Daamen et al., 2021), of which 10,463 had a non-zero COGS score in our dataset. Assuming that this set of DE genes is enriched for true causal loci, we performed a precision-recall analysis of these genes at a range of COGS thresholds between 0 and 1 (Supplementary Figure S8D). As expected, increasing the COGS threshold increased the enrichment for DE genes (a proxy for specificity or “precision”) among the prioritised candidates, but decreased their recall, as more DE genes ended up with subthreshold scores. Our predefined threshold of COGS score > 0.3 corresponded to a point at which the enrichment started to rise sharply (Supplementary Figure S8D), confirming that our choice of this threshold was reasonable for global downstream analyses. However, for more targeted selection of candidates (e.g., for small-scale perturbation experiments), using a higher COGS score threshold, which likely confers a higher specificity of the analysis at the expense of a lower sensitivity, may be warranted.

## Conclusion

The COGS pipeline combining Bayesian fine-mapping of GWAS signals with PCHi-C-based prioritisation has provided 251 putative genes associated with COVID-19 severity, most of which were not prioritised using the naive nearest-exon approach and the strategies used in the original COVID-19 HGI GWAS publication. Most of these genes have no known biological function in COVID-19 to date, but are enriched in pathways associated with inflammatory response to viral infection. In conjunction with complementary prioritisation approaches and targeted validation experiments (Cano-Gamez and Trynka, 2020), these data will help to understand and tackle COVID-19 pathology.

## Data Availability

The original contributions presented in the study are included in the article and Supplementary Material. Further inquiries can be directed to the corresponding authors.
