# Supplementary material for: Prioritisation of Candidate Genes Underpinning COVID-19 Host Genetic Traits Based on High-Resolution 3D Chromosomal Topology
Source: Front Genet. 2021 Oct 25;12:745672. doi: 10.3389/fgene.2021.745672 (PMC8573080; doi:10.3389/fgene.2021.745672)
Supplement: Supplementary file 4 [file DataSheet1.PDF]

# Prioritisation of candidate genes underpinning COVID-19 host genetic traits based on high-resolution 3D chromosomal topology

# Supplementary Figures

## Supplementary Figures 1-7: Manhattan plots

The Manhattan plots represent the genome-wide COGS scores across all cell types in the Javierre dataset (for GWASs B1, B2 and C2; **Fig. S1-3**) and the Ho dataset (for GWASs A2, B1, B2 and C2; **Fig. S4-7**). The top-scoring genes with scores > 0.3 are labelled in each locus. Multiple genes are labelled when there are several top-scoring genes with a very similar score, or lower-scoring genes with compelling biological functions. For simplicity, non-coding genes are not labelled, unless there are no prioritised protein-coding genes in the same locus.

Figure S1. COVID-19 HGI B1 gene-level COGS scores (all Javierre PCHi-C cell types)

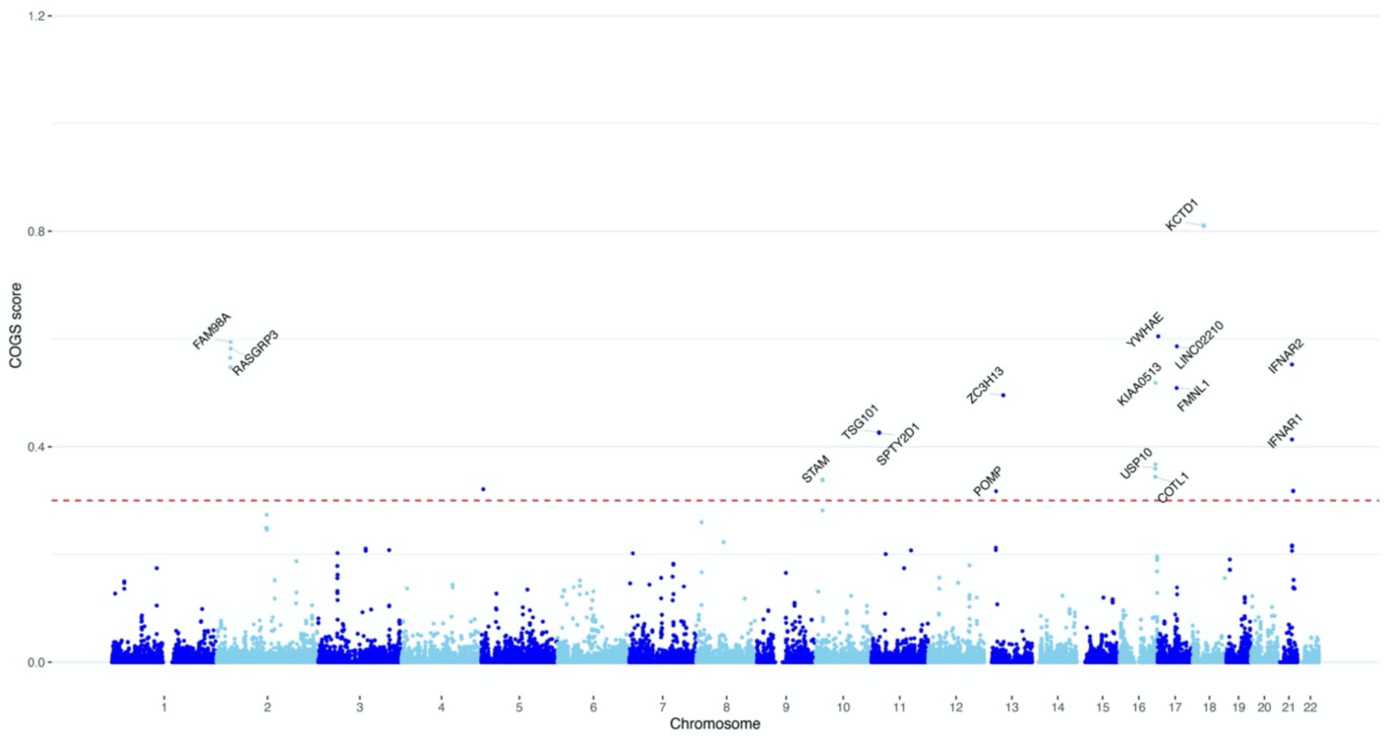

Figure S2. COVID-19 HGI B2 gene-level COGS scores (all Javierre PCHi-C cell types)

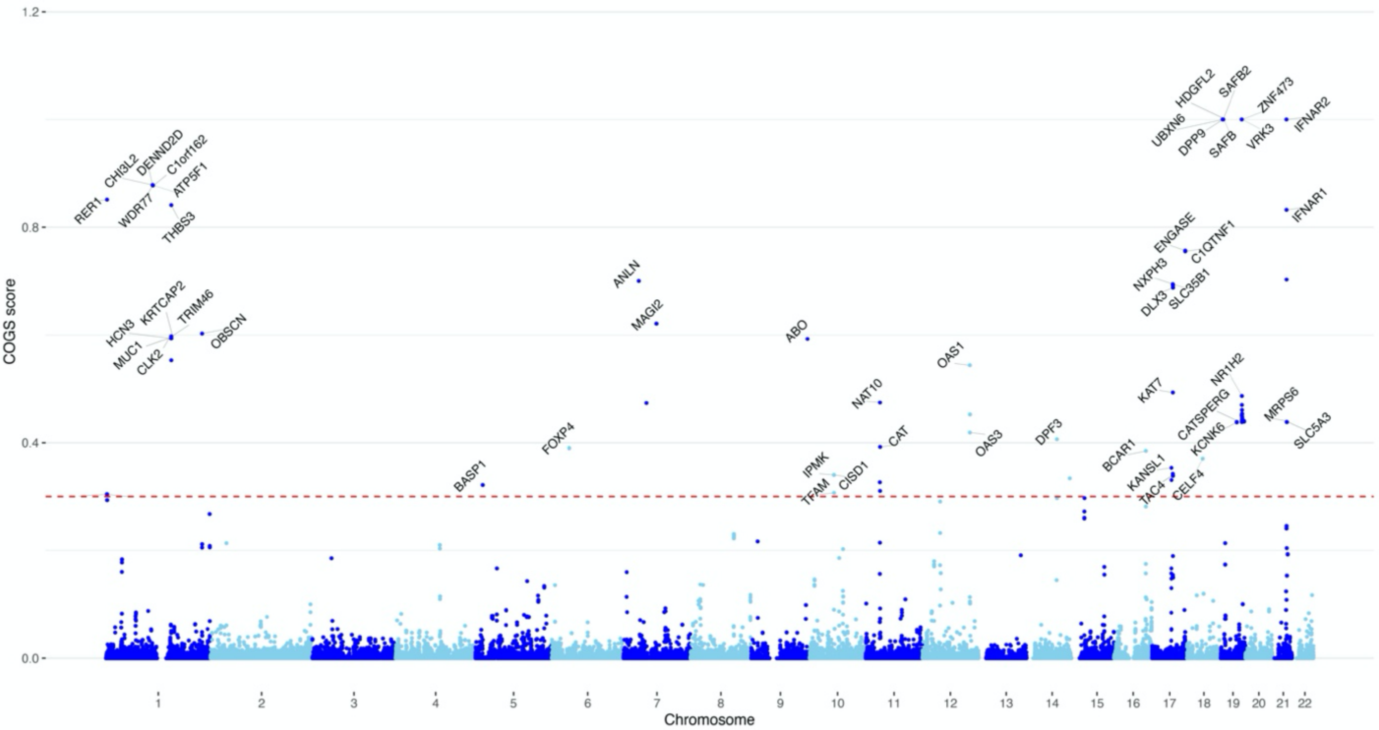


Figure S3. COVID-19 HGI C2 gene-level COGS scores (all Javierre PCHi-C cell types)

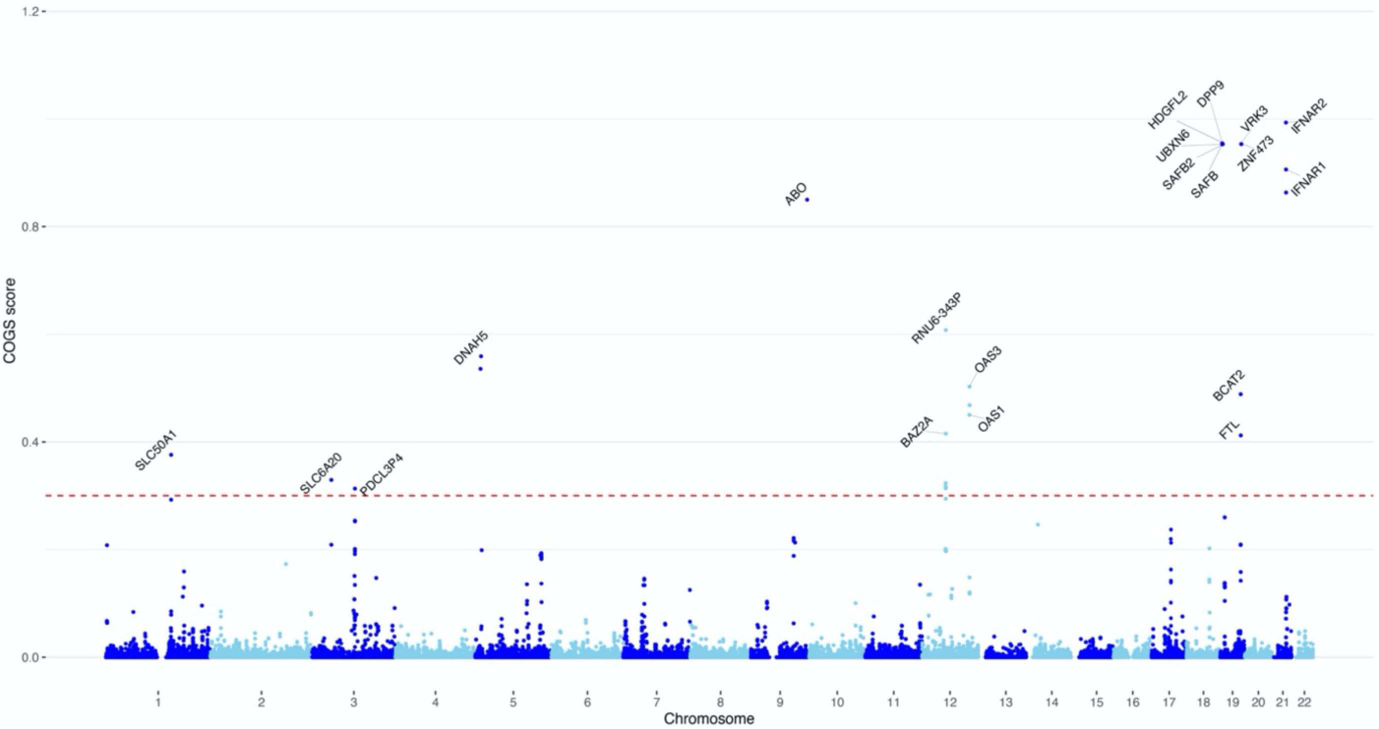

Figure S4. COVID-19 HGI A2 gene-level COGS scores (all Ho PCHi-C cell types)

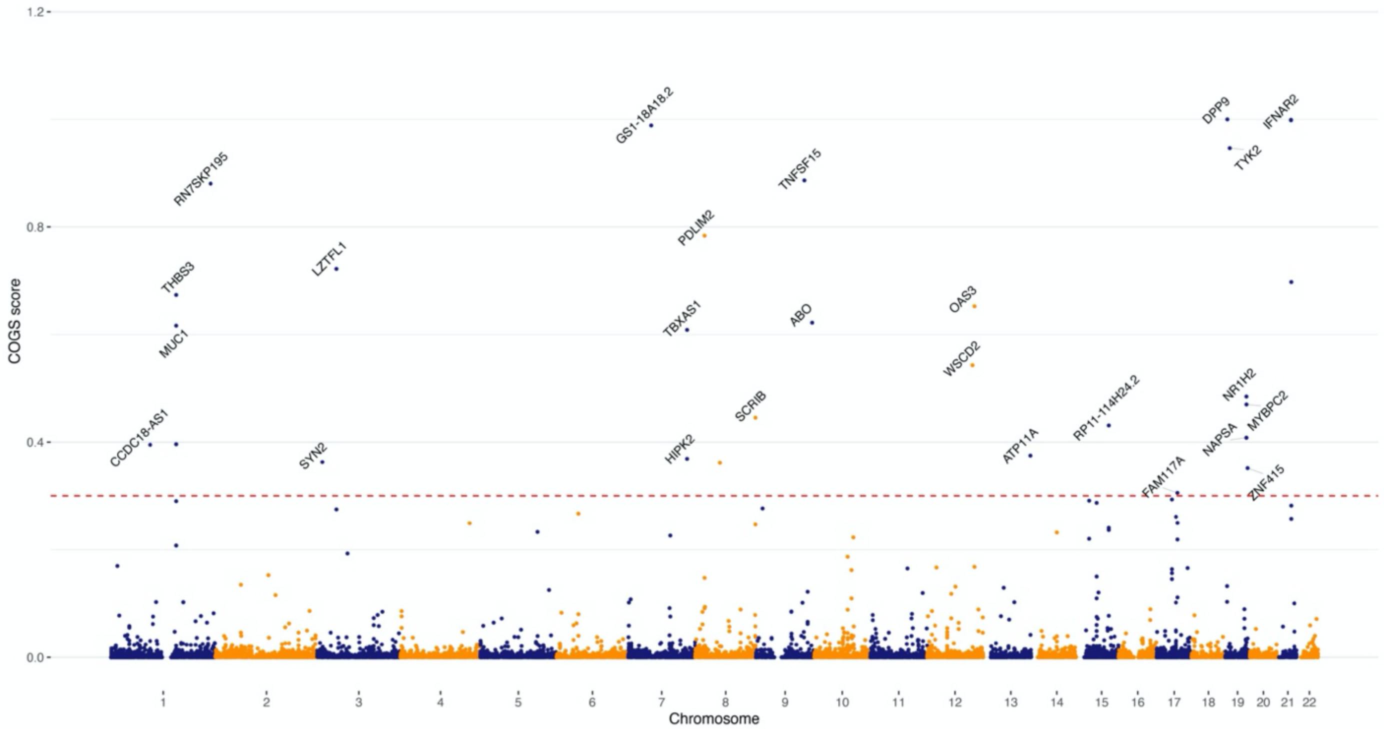


Figure S5. COVID-19 HGI B1 gene-level COGS scores (all Ho PCHi-C cell types)

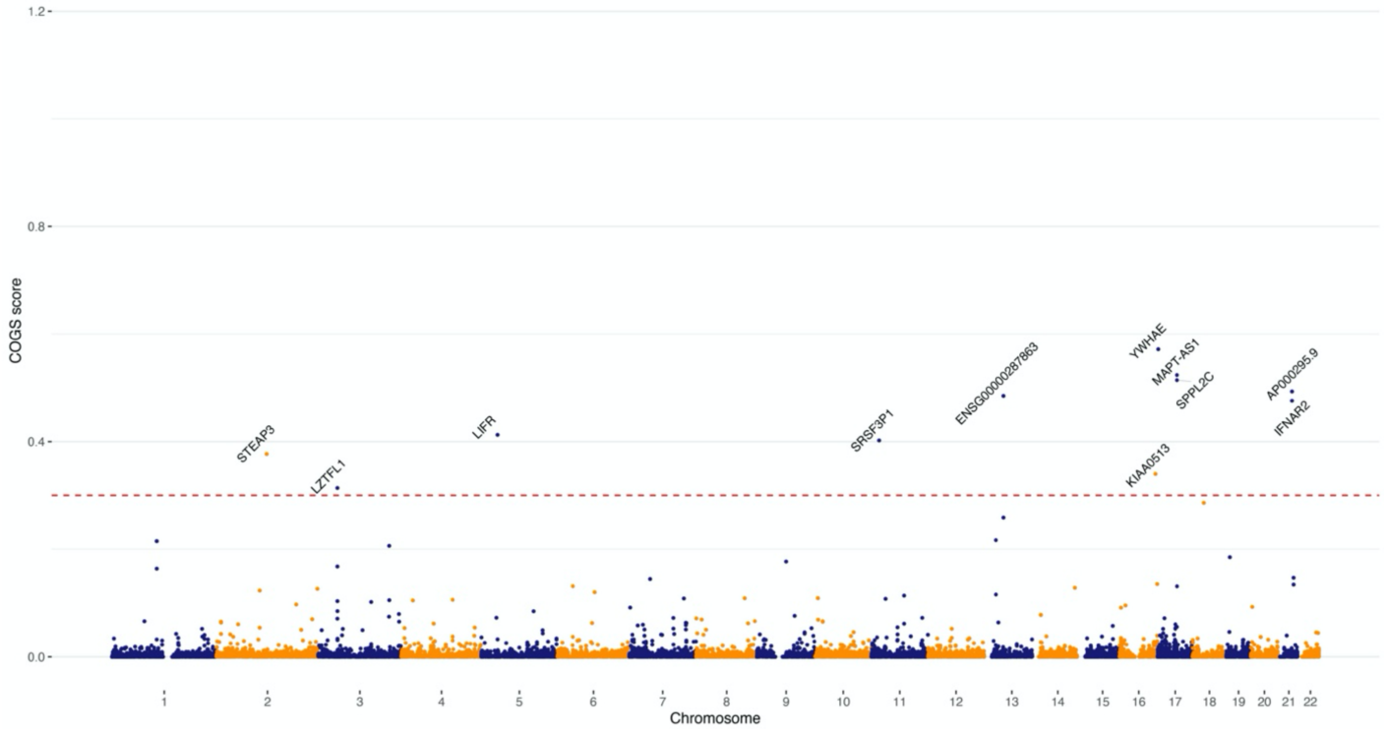

Figure S6. COVID-19 HGI B2 gene-level COGS scores (all Ho PCHi-C cell types)

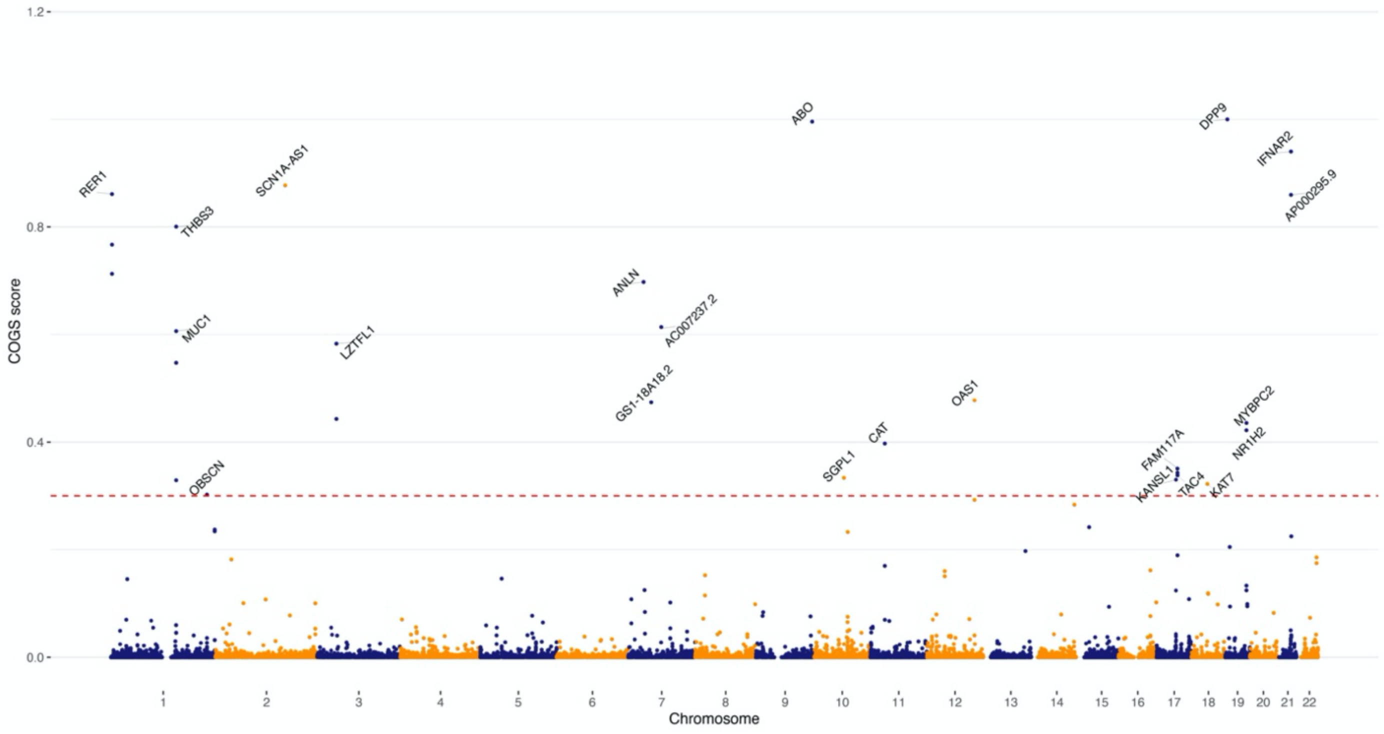


Figure S7. COVID-19 HGI C2 gene-level COGS scores (all Ho PCHi-C cell types)

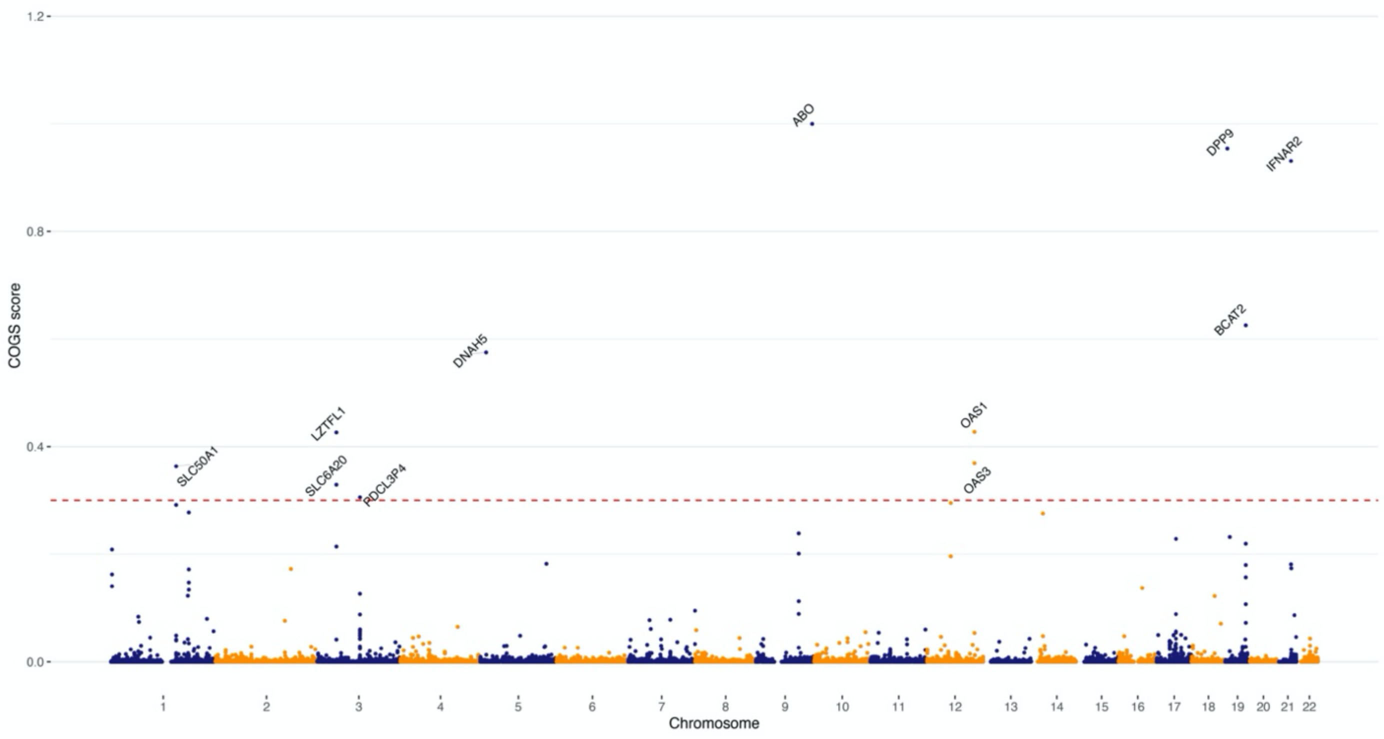

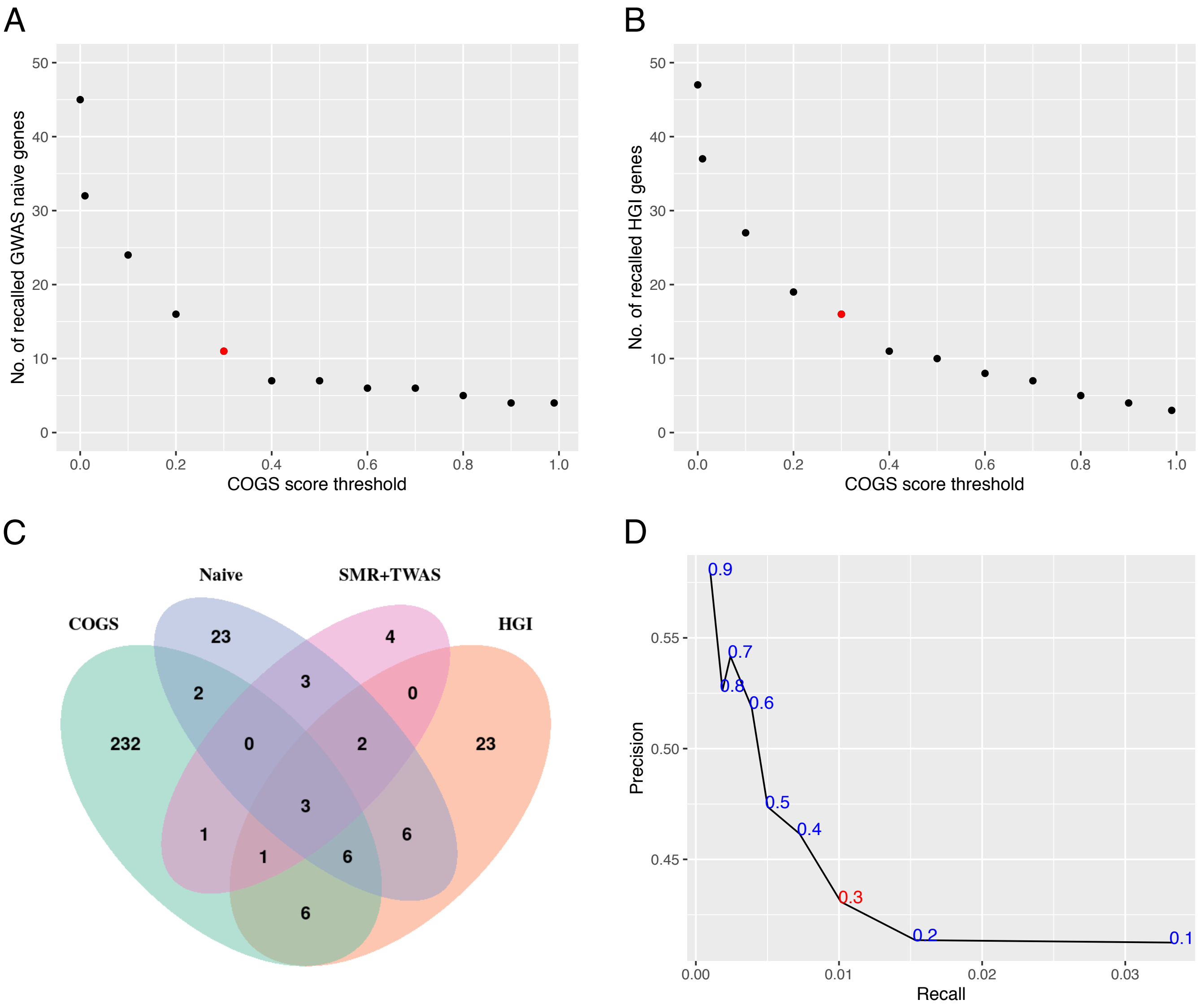


**Supplementary Figure 8: Comparison of COGS with alternative prioritisation approaches.** The number of recalled genes from the nearest-exon approach (A) and the HGI prioritisation approach (B) is shown for a range of COGS thresholds between 0 and 1. The red dot represents a COGS score threshold of 0.3, which was used for most downstream analyses in the present study. (C) Venn diagram representing the overlap of genes called by COGS (score > 0.3) and the three other approaches discussed in the study: naïve (nearest-exon), HGI (Host Genetics Initiative) and SMR + TWAS (Summary-based Mendelian Randomization + Transcriptome-wide Association Study). (D) Precision-recall curve for a set of differentially expressed genes in COVID-19 (Daamen et al., 2021) at a range of COGS score thresholds (shown by blue text, with the threshold of 0.3 highlighted in red).
